# Is Routine Gastroscopy/Colonoscopy Reasonable in Patients With Suspected Ovarian Cancer: A Retrospective Study

**DOI:** 10.3389/fonc.2021.608999

**Published:** 2021-07-01

**Authors:** Guochen Liu, Junping Yan, Shanshan Long, Zhimin Liu, Haifeng Gu, Hua Tu, Jundong Li

**Affiliations:** ^1^ Department of Gynecologic Oncology, State Key Laboratory of Oncology in South China, Collaborative Innovation Center for Cancer Medicine, Sun Yat-Sen University Cancer Center, Guangzhou, China; ^2^ Department of Laboratory Medicine, Guangdong Second Provincial General Hospital, Guangzhou, China

**Keywords:** ovarian cancer, gastroscopy, colonoscopy, ovarian metastasis, differential diagnosis

## Abstract

**Objective:**

To evaluate the value of routine preoperative gastroscopy/colonoscopy in patients with suspected ovarian cancer for differential diagnosis and judgment of bowel resection.

**Methods:**

All women diagnosed with suspected ovarian cancer who underwent gastroscopy/colonoscopy before surgery in our center were retrospectively identified. Gastroscopy/colonoscopy results and clinical pathology, imaging, and surgical findings were analyzed.

**Results:**

389 patients were included. Among them, 40 (including 13 gastric and 9 colonic malignancy) were ovarian metastasis. Compared with imaging, gastrointestinal endoscopy showed no statistical advantage in the specificity and sensitivity (99.4% vs. 99.7%, *P*=1.0; 55.0% vs. 45.2%, *P*=0.057; respectively). All patients with gastric/colonic cancer metastasize except for one had indicative imaging or tumor marker abnormalities. Three patients with colonic cancer metastases underwent optimal surgery and alive with no recurrence, the other 19 patients experienced palliative chemotherapy. There is no significant difference in the sensitivity of colonoscopy and imaging in predicting intestinal incision (61.5% vs. 43.8%, *P*=0.804), whereas the latter had higher specificity (87.8% vs. 74.3%, *P*=0.001).

**Conclusions:**

For patients with suspected ovarian cancer, the incidence of gastrointestinal metastases is low, routine gastroscopy/colonoscopy before treatment is less efficient. Gastroscopy/colonoscopy has limited power to predict the need for gastrointestinal resection before ovarian cancer surgery.

## Highlights

The value of routine preoperative gastroscopy/colonoscopy in patients with suspected ovarian cancer is unclear. Among patients with suspected ovarian cancer, routine gastroscopy/colonoscopy before treatment is less efficient. Gastroscopy/colonoscopy has limited power to predict the need for gastrointestinal resection before ovarian cancer surgery.

## Introduction

Early screening for ovarian cancer lacks effective methods, and many patients will resort to medical care because of symptomatic pelvic masses. According to the National Comprehensive Cancer Network guideline, patients with newly diagnosed pelvic masses suspected of ovarian cancer should be considered as candidates for gastroscopy/colonoscopy. This strategy is mainly due to the following considerations: First, it has been reported that about 3.2%~7.0% of ovarian tumors are metastasized from the stomach or colon, namely the Krukenberg tumor (1–3). For these patients, therapeutic decisions should be made by surgical oncologists rather than gynecologists. Second, ovarian cancer is prone to disseminate in the abdominal cavity, in which the digestive tract is most vulnerable, and preoperative evaluation is quite important. According to the literature, approximately 20% of patients underwent gastrointestinal procedures during cytoreductive surgery for ovarian cancer (4–7). Findings from gastrointestinal endoscopy will allow more sufficient preoperative preparation for these patients. Mucosa involvement and loss of elasticity are signs of tumor invasion that may require bowel resection.

However, gastrointestinal endoscopy also has several disadvantages, including causing discomfort to the patients, increased medical costs, delay in treatments, and the risks of gastrointestinal perforation, bleeding, and cardiovascular and cerebrovascular accidents, etc. Moreover, employing patient symptoms, physical examination, tumor marker examination, preoperative imaging evaluation, and puncture pathology, it is also possible to indirectly determine the source of the tumor or whether there is gastrointestinal involvement. The small intestine is also frequently involved in ovarian cancer, for which colonoscopy has limited detection capability (6). In clinical practice, we found that few patients had changed their diagnoses or established treatment strategies due to the findings from gastrointestinal endoscopy. Therefore, it is questionable whether routine gastrointestinal endoscopy is a rational strategy for patients with suspected ovarian cancer at initial diagnosis.

In our center, gastroscopy/colonoscopy has been routinely performed in the majority of patients who were suspected to have ovarian cancer. Based on a large number of screened patients, we conducted a retrospective study to evaluate the rationality of routine gastroscopy/colonoscopy before treatment for patients suspected of ovarian cancer.

## Methods

With the approval of the Research Ethics Committee of the Sun Yat‐sen University Cancer Center, we retrospectively collected the information of patients diagnosed with suspected ovarian cancer by imaging examinations who underwent gastroscopy/colonoscopy before treatment in our center from November 1, 2016, to October 29, 2019. Pelvic mass biopsy or surgical pathology results in our hospital were required. Patients with a history of gastrointestinal cancer or ovarian cancer, or who had a definite pathological diagnosis before the gastrointestinal examination, or did not undergo imaging (BUS/CT/MRI/PET-CT) examination, or with previous gastric or intestinal surgery, or suffering from chronic intestinal diseases were excluded. The endoscopy system was searched to obtain the gastroscopy/colonoscopy results. Clinicopathological information includes age, symptoms, physical examination, tumor marker values, preoperative imaging results, surgical records, and postoperative pathological results, which were obtained from the hospital information system (HIS) system. We reviewed the patients’ imaging reports in the PACS system to determine the possible source of pelvic masses and whether it involves the intestine, the most common abnormal findings indicating bowl infiltration by preoperative imagings were compression of the bowel, stricture, areas of constricture, and/or mucosal ulceration. If the source of the tumor cannot be determined, it was recorded as unknown.

SPSS software (SPSS Inc., Chicago, IL, version 19) was used for data analysis. We analyzed the diagnostic efficacy of gastrointestinal symptoms, tumor indicators, unilateral and bilateral accessory lesions, imaging examination, and gastroscopy/colonoscopy for metastatic ovarian tumors. We used the ROC curve to separately analyze the discrimination of different tumor markers for ovarian metastases. The Mann-Whitney U rank-sum test and the Wald chi-square (χ^2^) test was used for comparison of two sets of quantitative data and categorical parameters, respectively. The McNemar test was used to compare the difference between specificity and sensitivity. All P values were two-sided, and P values of less than .05 were considered statistically significant.

## Results

**Figure 1:**
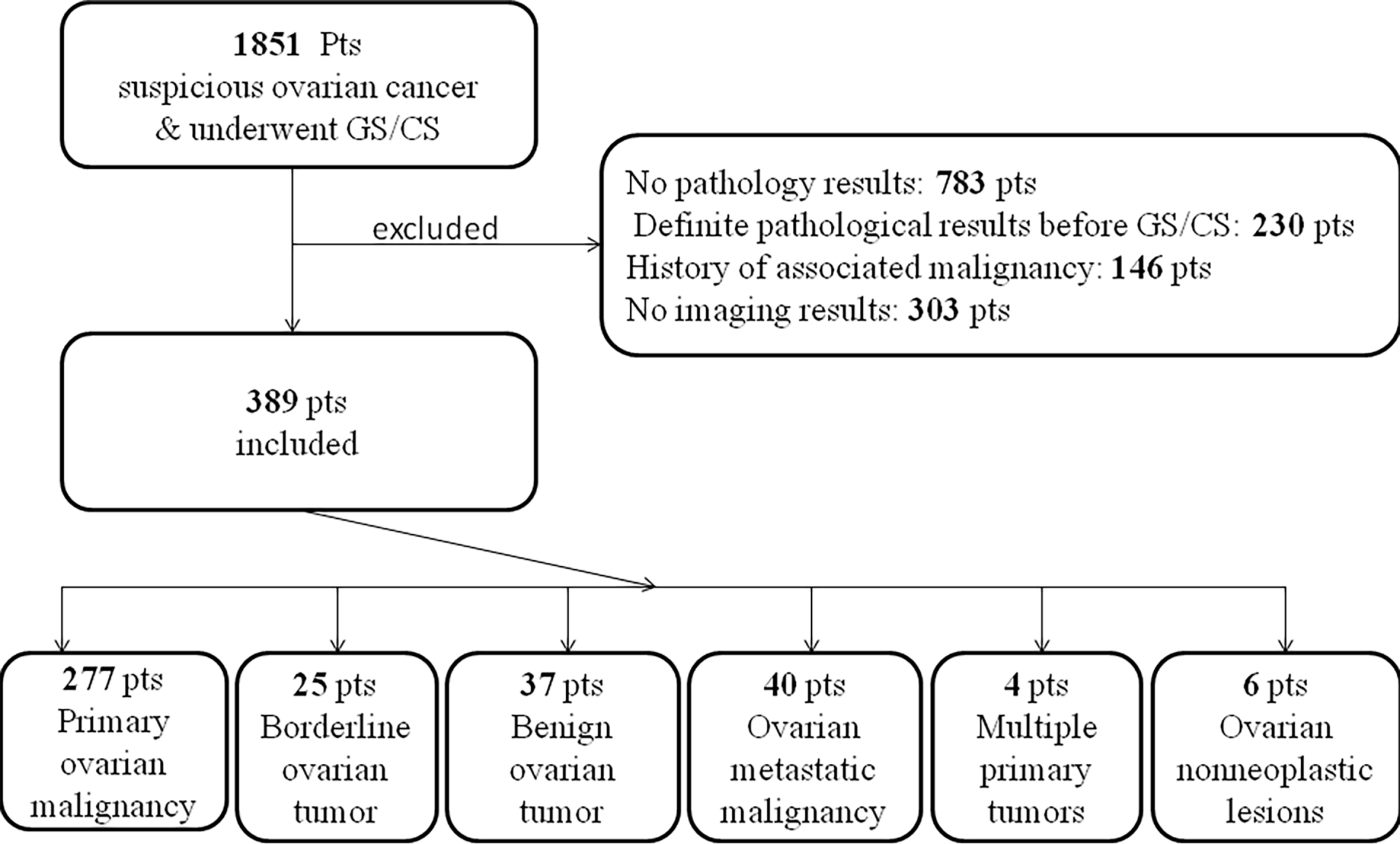
Flow diagram of patient selection and final diagnosis.

After screening 1851 patients (**Figure 1**), a total of 389 eligible patients were included in the study, of which 302 patients had gastroscopy and colonoscopy at the same time, 46 had gastroscopy only, and 41 had colonoscopy only. The patients’ basic information is shown in **Table 1**.

**Table 1:** Demographic and clinical-pathological characteristics.

Variables	Group		*P*
	Primary ovarian tumor (n=339) No.(%)	Ovarian metastatic tumor (n=40) No.(%)	
Age, y			0.337†
Median	51	48	
Range	18~85	21~78	
Side			0.002*
Unilateral	219(64.6)	16(40.0)	
Bilateral	120(35.4)	24(60.0)	
Digestive symptoms			0.074*
Negative	143(42.2)	11(27.5)	
Positive	196(57.8)	29(72.5)	
CA125			
<=35 U/ml	63(18.6)	15(37.5)	0.003*
>35 U/ml	271(79.9)	23(57.5)	
Unknown	5(1.5)	2(5.0)	
CEA			
<=5 ng/ml	284(83.8)	17(42.5)	<0.001*
>5 ng/ml	43(12.7)	23(57.5)	
Unknown	12(3.5)	0(0)	
CA199			
<=35 U/ml	241(71.1)	21(52.5)	0.020*
>35 U/ml	88(26.0)	17(42.5)	
Unknown	10(2.9)	2(5.0)	
HE4			
<=90pmol/L	103(30.4)	23(57.5)	<0.001*
>90pmol/L	193(56.9)	11(27.5)	
Unknown	43(12.7)	6(15.0)	
CA125/CEA			
<=25	81(23.9)	24(60.0)	<0.001*
>25	246(72.6)	14(35.0)	
Unknown	12(3.5)	2(5.0)	

†P values were calculated using a two-sided Mann-Whitney U rank-sum test.

*P values were calculated using a two-sided Wald χ^2^ test.

Of the 348 patients who had a gastroscopy, 13 had pathologically confirmed primary malignant tumors of the stomach (11 biopsies confirmed gastric cancer, 2 cases were gastric lymphoma), 45 were normal, 37 had polypus confirmed by biopsy, 6 had external pressure lesions, 7 cases had gastric inflammation with gastric ulcer, 10 cases had gastritis and polyps at the same time, 230 cases had stomach inflammation (of which 220 cases had chronic non-atrophic gastritis, accounting for 95.7%).

Of the 343 patients who underwent colonoscopy, 10 cases had biopsy-confirmed bowel cancer, 162 had no abnormalities, 78 had polyps, 21 had inflammation and polyps, 72 had extrinsic compression or infiltration (of which 2 had failed colonoscopy due to external pressure of the tumor). It is worth noting that of the 2 patients with multiple intestinal polyps, 1 patient was diagnosed with FAP (familial adenomatous polyposis) and the other was considered P-J (Peutz-Jeghers syndrome) syndrome.

Confirmed by biopsy or postoperative pathology, as illustrated in **Figure 1**, among all patients with the initial diagnosis of suspected ovarian cancer, 277 cases were ovarian primary malignant tumors, 25 cases were ovarian primary borderline tumors and 37 cases were ovarian primary benign tumors. 40 cases were ovarian metastatic tumors (11 gastric cancer, 9 colonic cancer, 8 appendix mucinous tumor, 2 gastric lymphomas, 2 endometrial cancer, 2 pancreatic cancer, 1 cholangiocarcinoma, 1 peritoneum Malignant mesothelioma, 1 small intestinal stromal tumor, 1 liver cancer, 1 cervical cancer, 1 unclear primary pathology). 4 cases of multiple primary tumors (1 case of ovarian cancer with gallbladder cancer, 1 case of ovarian cancer sarcoma with appendix mucinous tumor, 1 case of sigmoid colon cancer with ovarian cancer, and 1 case of lymphoma with ovarian teratoma). 6 cases were ovarian non-neoplastic lesions (2 inflammatory lesions, 2 subuterine fibroids, 1 small intestinal stromal tumor, and 1 ovarian tuberculosis).

Compared with primary ovarian tumors, patients with ovarian metastases are mostly bilateral lesions (60% vs. 35.4%, *P*=0.002), ovarian cancer indicators CA125 (37.5% vs. 18.6%, *P*=0.003) and HE4 are more normal (57.5% vs. 30.4%, *P*<0.001), gastrointestinal cancer indicators CEA (57.5% vs. 12.7%, *P*<0.001) and CA199 (42.5% vs. 26%, *P*=0.020) are more abnormal, and the ratio of CA125/CEA less than 25 is higher (60.0% vs. 23.9%, *P*<0.001). Besides, although there is no statistical difference, patients with ovarian metastases are more likely to have gastrointestinal symptoms (72.5% vs. 57.8, *P*=0.074). Among them, abdominal distension and abdominal pain are the most common (24/40), and 2 patients each have black stools and diarrhea, 1 patient developed constipation (**Table 1**).

**Figure 2:**
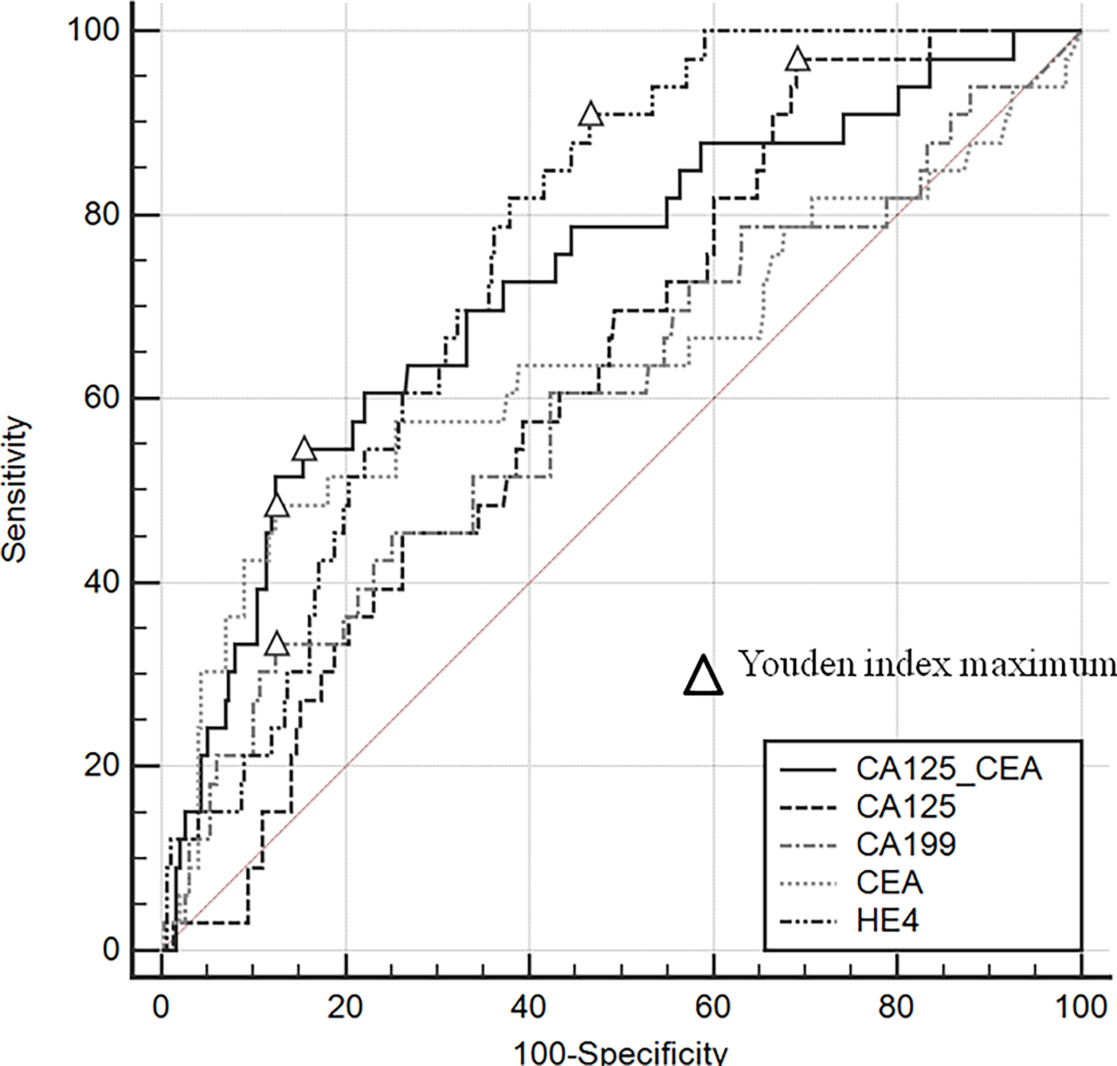
The ROC curves of different tumor markers.

Gastrointestinal endoscopy and imaging had a high diagnostic efficacy (94.9% vs. 94.4%, respectively) for ovarian metastases, (**Table 2**). There was no statistical difference between the specificity and sensitivity of the two methods (99.4% vs. 99.7, *P*=1.0; 55.0% vs. 45.2%, *P*=0.057). The area under the curve (AUC) in descending order were: HE4(0.756), CA125/CEA(0.730), CEA(0.642), CA125(0.629), CA199(0.602) (**Figure 2**). Different from previous research (8), we found that when CA125/CEA=10.57, the Youden Index value was the largest, and the specificity and sensitivity were 87.83% and 55.26% respectively.

**Table 2:** The diagnostic value of ovarian metastatic carcinoma by different criteria.

Criterion	Specificity(%)	Sensitivity(%)	PPV(%)	NPV(%)	DE(%)
Gastroscopy/colonoscopy	99.4	55.0	91.7	95.1	94.9
Imaging scan	99.7	45.2	93.3	94.5	94.4
Digestive symptoms	42.2	72.5	12.9	92.9	45.4
CA125	80.6	35.0	17.5	91.3	75.8
CEA	87.0	51.3	30.8	94.0	83.3
CA199	75.7	63.2	22.2	94.9	74.4
HE4	62.8	60.5	17.0	92.6	62.5
Bilateral adnexal lesion	54.3	66.7	15.3	93.0	55.7
CA125/CEA	74.9	63.2	22.0	94.8	73.7

PPV, positive predictive value; NPV, negative predictive value; DE, diagnostic efficiency. Digestive symptoms include: abdominal distension, abdominal pain, diarrhea, constipation, vomiting, hematochezia, melena.

Imaging scan include: color ultrasound, CT, MRI, PET-CT.

Patients meet one of the following criterions were considered as ovarian metastatic carcinoma: positive gastrointestinal symptoms, CA125<35U/ml, CEA>5ng/ml, CA199>35U/ml, HE4<90pmol/L, bilateral adnexal lesion or CA125/CEA<25.

**Figure 3:**
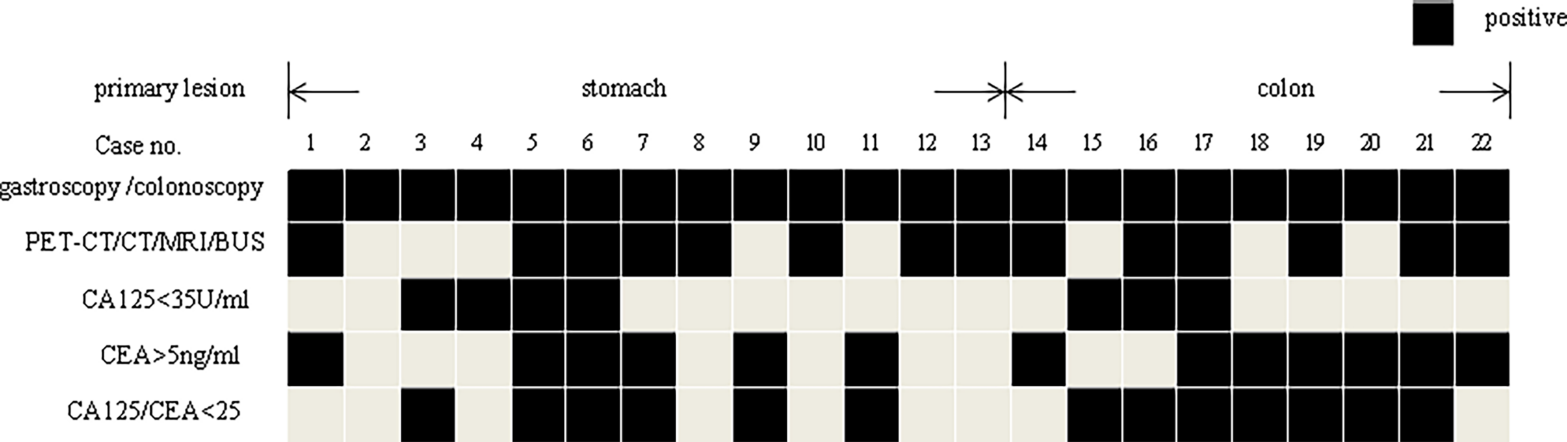
The gastroscopy/colonoscopy, imaging, and tumor marker results of the patients’ metastasis from stomach or colon.

**Figure 3** displays the examination results of 13 patients with gastric metastases and 9 colonic metastases. Except for one patient with gastric cancer who has no corresponding imaging or tumor marker abnormalities (Case 2), all the remaining patients had an indication tumor maker or imaging.

Thirteen patients with malignant tumors of the primary stomach, including 2 cases with gastric lymphoma, all received matching chemotherapy after obtaining pathological evidence for metastasis by ultrasound-guided accessory tumors puncture. Three patients with colon metastases underwent surgical treatment after ruled out other sites of metastasis and received chemotherapy for colon cancer subsequently. Fortunately, all three patients survived without tumors at the last follow-up. The other 6 cases of colon cancer underwent ultrasound-guided puncture of the adnexal mass. After the metastasis was confirmed, all patients received chemotherapy according to the corresponding chemotherapy regimen. The treatment and prognosis of the patient are shown in **Table 3**.

**Table 3:** The treatment and prognosis of the 22 ovarian metastases from the stomach or colon.

Case no.	Gastroscope/colonoscopy Correct diagnosis	Initial primary treatment plan	Actual primary treatment	Outcome at last Surveillance	OS (months)
1	Gastric cancer	Surgery	Chemotherapy	DOD	16.7
2	Gastric cancer	Surgery	Chemotherapy	DOD	16.3
3	Gastric cancer	Surgery	Chemotherapy	AWD	16.4
4	Gastric cancer	Surgery	Chemotherapy	DOD	5.2
5	Gastric cancer	Surgery	Chemotherapy	AWD	18.5
6	Gastric cancer	Surgery	Chemotherapy	NED	35.0
7	Gastric cancer	Surgery	Chemotherapy	AWD	10.0
8	Gastric cancer	Surgery	Chemotherapy	DOD	21.9
9	Gastric cancer	Surgery	Chemotherapy	DOD	12.5
10	Gastric cancer	Surgery	Chemotherapy	NED	19.8
11	Gastric cancer	Surgery	Chemotherapy	Unknown	Unknown
12	Gastric lymphoma	Surgery	Chemotherapy	NED	3.2
13	Gastric lymphoma	Surgery	Chemotherapy	AWD	24.2
14	Sigmoid colon cancer	Surgery	Chemotherapy	NED	10.7
15	FAP, ascending colon cancer	Surgery	Chemotherapy	DOD	28.3
16	Ascending colon cancer	Surgery	Surgery	NED	5.03
17	Sigmoid colon cancer	Surgery	Chemotherapy	NED	10.0
18	Ascending colon cancer	Surgery	Surgery	NED	17.9
19	Sigmoid colon cancer	Surgery	Surgery	NED	31.9
20	Sigmoid colon cancer	Surgery	Chemotherapy	NED	1.3
21	Sigmoid colon cancer	Surgery	Chemotherapy	Unknown	Unknown
22	Descending colon cancer	Surgery	Chemotherapy	DOD	25.9

OS, overall survival; DOD, die of disease; AWD, alive with disease; NED, no evidence of disease.

Of the 277 patients with ovarian cancer who underwent surgery, 32 underwent colon surgery (2 right hemicolectomies, 9 sigmoidectomies, and 21 Dixon), no patient underwent gastric surgery at the same time. **Table S1** shows the predictive value of gastroscopy/colonoscopy and imaging in bowel resection. If the intestinal pressure and invasion were considered as signs of intestinal resection, there was no significant difference in the sensitivity of colonoscopy and imaging to the prediction of intestinal incision compared between the two groups (61.5% vs. 43.8%, *P*=0.804), however, the imaging’s specificity is higher (87.8% vs. 74.3%, P=0.001). It is noteworthy that the above gastrointestinal surgery mentioned does not include appendix and small bowel surgery, one patient each had preoperative imaging indicated invasion of the appendix and small intestine underwent surgery on the corresponding part.

## Discussion

There is no clear recommendation for the application of gastroscopy/colonoscopy for patients with suspected ovarian cancer in the current guidelines. The choice of gynecologists can be very varied when treating these patients.

In our study, only 5.7% (22/389) of the patients had gastrointestinal metastases. It is worth noting that, except for one patient, the remaining patients all had imaging or tumor marker indicators. If the gastrointestinal examination is performed only on patients with imaging or tumor marker indicators, 62.2% (242/389) of patients can be spared from an unnecessary gastrointestinal examination, which means great medical cost savings. Therefore, risk-adapted gastrointestinal endoscopy may be a more reasonable strategy for patients suspected of ovarian cancer.

Although one patient got a false-negative results in a gastrointestinal examination, neither her treatment nor prognosis had been impaired. After pathological confirmation of gastrointestinal cancer, salvage treatment of gastrointestinal tumors were given. In the present study, most patients showing positivity in gastrointestinal endoscopy changed their treatment strategies and abandoned the planned surgeries, according to the current recommendations for metastatic gastrointestinal cancer. However, surgery can yet be regarded as another choice for these patients. Many studies had revealed that for patients with primary bowel cancer and ovarian metastasis, optimal cytoreductive surgery could also bring a favorable prognosis (9, 10). Three patients in our study with bowel cancer achieved long-term survival without recurrence after surgery, which supports the above findings. Similarly, for patients with gastric cancer and ovarian metastasis, Cheong et al. found that the removal of metastases can improve the prognosis of patients (11). Furthermore, a Korean study validated that the removal of ovarian metastases plus palliative chemotherapy provided a better prognosis than palliative chemotherapy alone (12). In light of the above evidence, if the surgery can achieve satisfactory resection, it will not delay the treatment of the patients or affect their prognosis. For patients who cannot obtain optimal resection, the removal of ovarian masses can also be beneficial, since these metastases were usually chemotherapy-resistant (9, 13–17). Moreover, ovarian metastatic tumors are relatively large, with the average sizes of 9-12 cm in previous studies (2, 18). Palliative resection of these tumors could relieve symptoms such as abdominal distension and pain (10). In short, it is still of diagnostic and therapeutic value to perform surgery for patients with primary gastrointestinal cancer and ovarian metastases. Accordingly, the value of gastrointestinal endoscopy as a routine procedure to distinguish these patients from those with ovarian cancer is very limited.

Moreover, unlike imaging examinations, gastrointestinal endoscopy cannot find primary lesions beyond the stomach and large intestine, such as appendiceal cancer. However, studies have shown that ovarian metastases more commonly arise from the appendix than the stomach (2). This further reduces the value of routine gastrointestinal endoscopy. In the present study, metastases originating in the stomach were more common. This discrepancy may be accredited to a higher incidence of gastric cancers in China.

When there is a pelvic mass whose origin cannot be defined, patients usually need to receive fine-needle aspiration of pelvic masses to obtain histopathological evidence. Due to the risks of needle path metastasis and the difficulty of pathological diagnosis based on small-volume tissue, surgical exploration may be a better choice, which is also necessary for patients with ovarian cancer requiring neoadjuvant chemotherapy. When a pathological diagnosis is in doubt, then gastrointestinal examination is performed to exclude gastrointestinal metastasis, which can also avoid a considerable number of patients receiving gastrointestinal endoscopy. Following the recommendations of the guidelines, obtaining accurate histological evidence before chemotherapy in such patients will prevent improper chemotherapy.

Furthermore, similar to the conclusions of previous studies (4, 5), in patients receiving cytoreductive surgery for ovarian cancer, compared with imaging, gastroscopy/colonoscopy cannot predict gastric or large intestine resection well before surgery, nor can it predict the possibility of small intestine and appendectomy. The proportion of these patients in ovarian cancer reduction surgery is not low (6).

To our knowledge, the present study is the largest retrospective study with the largest sample size to evaluate the value of routine preoperative gastroscopy/colonoscopy in patients with suspected ovarian cancer. However, this study is a retrospective one, which has several limitations. Firstly, the study included a small number of patients with benign and non-ovarian tumors, for whom the gastroscopy/colonoscopy is not generally necessary. Compared with patients with primary ovarian cancer, patients with metastatic ovarian tumors accounted for a significantly lower proportion in this study, which might have impaired the statistical power of the results. This is mainly due to the inclusion of consecutive unselected patients, which was designed to reflect the actual clinical practice as much as possible. Secondly, the physical examination was not included as an indicator because the results of medical examinations by different doctors are usually inconsistent and subjective. Besides, considering that rectal cancer accounts for a higher proportion of colorectal cancer and the convenience of physical examination to determine the possibility of rectal resection, the importance of colonoscopy may be further weakened. Thirdly, whether neoadjuvant chemotherapy or satisfactory reduction during surgery had affected the proportion of bowel resection and thereby affected our evaluation on gastrointestinal endoscopy remains unclear, Due to the retrospective nature and the relatively small sample size, we did not discuss it, which requires further investigation by future studies. Finally, there is no ideal way of predicting prior to the operation whether a resection of bowel is necessary, or the extent of the bowel resection even if the imaging is considered abnormal. Similarly, a normal preoperative imagings likewise did not preclude a bowel resection (19).

## Conclusion

Among patients with suspected ovarian cancer, the proportion of gastrointestinal metastases is low, and the efficiency of routine gastroscopy/colonoscopy before treatment is quite lacking. It seems more reasonable to adopt a risk-adapted gastroscopy/colonoscopy strategy based on imaging examination and tumor markers. Before ovarian cancer surgery, gastrointestinal endoscopy has limited power to predict the need for gastrointestinal resection.

## Data Availability Statement

The original contributions presented in the study are included in the article/**Supplementary Material**. The authenticity of this article has been validated by uploading the key raw data onto the Research Data Deposit public platform (www.researchdata.org.cn), with the approval RDD number RDDA2021002026. Further inquiries can be directed to the corresponding authors.

## Author Contributions

GL: Data curation, investigation, writing - original draft, and writing - review and editing. JY: Data curation, investigation, writing – original draft, and formal analysis. SL: Data collection, investigation, validation, and methodology. ZL: Data curation and formal analysis. HG: Data curation and formal analysis. HT: Investigation, methodology, project administration, supervision, validation, writing - original draft, and writing - review and editing. JL: Investigation, methodology, project administration, supervision, validation, writing - original draft, and writing - review and editing. All authors contributed to the article and approved the submitted version.

## Funding

This work was supported by funds from the Nature Science Foundation of China (No. 81802615).

## Conflict of Interest

The authors declare that the research was conducted in the absence of any commercial or financial relationships that could be construed as a potential conflict of interest.

## Supplementary Material

Supplementary Table 1 The predictive value of gastroscopy/colonoscopy and imaging of gastrointestinal surgery performed as a part of surgical debulking for patients with primary ovarian cancer.
