# Supplementary material for: Is Routine Gastroscopy/Colonoscopy Reasonable in Patients With Suspected Ovarian Cancer: A Retrospective Study
Source: Front Oncol. 2021 Jul 1;11:608999. doi: 10.3389/fonc.2021.608999 (PMC8281959; doi:10.3389/fonc.2021.608999)
Supplement: Supplementary Table 1 — The predictive value of gastroscopy/colonoscopy and imaging of gastrointestinal surgery performed as a part of surgical debulking for patients with primary ovarian cancer. [file DataSheet_1.zip › Table S1.DOCX]

| Table S1. The predictive value of gastroscopy/colonoscopy and imaging of gastrointestinal surgery performed as a part of surgical debulking for patients with primary ovarian cancer | | | | | |
| --- | --- | --- | --- | --- | --- |
| Preoperative examination | Gastrointestinal affected types | Stomach surgery  (n of pts.) | Colorectal surgery  (n of pts.) | No stomach/ colorectal surgery  (n of pts.) |  |
| Gastroscope | Infiltration | 0 | N/A | 0 |  |
|  | Compression | 0 | N/A | 6 |  |
|  | Normal | 0 | N/A | 243 |  |
| Colonoscopy | Infiltration | N/A | 3 | 7 |  |
|  | Compression | N/A | 13 | 49 |  |
|  | Normal | N/A | 10 | 162 |  |
| Imaging scan | Infiltration | 0 | 14 | 30 |  |
|  | Normal | 0 | 18 | 215 |  |

n, number; pts, patients.
